# Pre-Exposure of *Mycobacterium tuberculosis*-Infected Macrophages to Crystalline Silica Impairs Control of Bacterial Growth by Deregulating the Balance between Apoptosis and Necrosis

**DOI:** 10.1371/journal.pone.0080971

**Published:** 2013-11-22

**Authors:** Leslie Chávez-Galán, Lucero A. Ramon-Luing, Luis Torre-Bouscoulet, Rogelio Pérez-Padilla, Isabel Sada-Ovalle

**Affiliations:** 1 Laboratory of Integrative Immunology, National Institute of Respiratory Diseases Ismael Cosío Villegas, Mexico City, Mexico; 2 Department of Respiratory Physiology, National Institute of Respiratory Diseases Ismael Cosío Villegas, Mexico City, Mexico; Institut de Pharmacologie et de Biologie Structurale, France

## Abstract

Inhalation of crystalline silica (CS) particles increases the risk of pulmonary tuberculosis; however, the precise mechanism through which CS exposure facilitates *Mycobacterium tuberculosis* (Mtb) infection is unclear. We speculate that macrophage exposure to CS deregulates the cell death pathways that could explain, at least in part, the association observed between exposure to CS and pulmonary tuberculosis. We therefore established an *in vitro* model in which macrophages were exposed to CS and then infected with Mtb. Expression of surface markers was analyzed by flow cytometry, JNK1/2, ASK1, caspase 9, P-p38, Bcl-2 and Mcl-1 were analyzed by Western blot, and cytokines by ELISA. Our results show that exposure to CS limits macrophage ability to control Mtb growth. Moreover, this exposure reduced the expression of TLR2, Bcl-2 and Mcl-1, but increased that of JNK1 and ASK1 molecules in the macrophages. Finally, when the pre-exposed macrophages were infected with Mtb, the concentrations of TNFα, IL-1β and caspase-9 expression increased. This pro-inflammatory profile of the macrophage unbalanced the apoptosis/necrosis pathway. Taken together, these data suggest that macrophages exposed to CS are sensitized to cell death by MAPK kinase-dependent signaling pathway. Secretion of TNF-α and IL-1β by Mtb-infected macrophages promotes necrosis, and this deregulation of cell death pathways may favor the release of viable bacilli, thus leading to the progression of tuberculosis.

## Introduction

Tuberculosis is an infectious disease caused by *Mycobacterium tuberculosis* (Mtb). According to the World Health Organization (WHO), in 2011 there were an estimated 8.7 million incident cases of tuberculosis and 1.4 million people died of this disease [1,2]. Exposure to significant amounts of CS through inhalation leads to lung inflammation and the development of silicosis and/or silico-tuberculosis [3-5]; however, the mechanisms through which CS exposure alters macrophage function have not been fully elucidated.

Alveolar macrophages (AM) are critical components of the innate and adaptive immune systems, and the prototypical host cell for diverse pathogens, including Mtb and many airborne particles [6,7]. Interaction of AM with CS particles has long been studied using *in vitro* and *in vivo* toxicity models [8-10]. Macrophages and alveolar type II cells exposed to CS increase cellular stress, the secretion of reactive oxygen species (ROS) and pro-inflammatory cytokines, such as IL-1β, by cells undergoing apoptosis [11-15]. Cellular stress is followed by phosphorylation of the mitogen-activated protein kinase (MAPK) family members, including apoptosis signal-regulating kinase 1 (ASK1), c-Jun N-terminal kinase (JNK) and p38 proteins [16-18]. In addition, active secretion of IL-1β that is partially dependent on caspase-1 activity, which in turn requires the assembly and activation of the Nalp3 inflammasome, is a new, innate immune mechanism that contributes to mycobacterial clearance by promoting phagolysosomal maturation and pyroptosis of Mtb-infected macrophages [19-21]. 

Mtb has developed many strategies to evade the host’s protective defenses [22]. Manipulation of phagosome maturation and deregulation of cell death pathways are well-known mechanisms that facilitate survival of the bacilli in their niche [23,24]. For example, virulent Mtb strains stimulate cell death by necrosis, allowing viable bacilli to escape from adjacent cells. However, precisely how the immune-physiological function of the macrophage is altered when exposed to CS particles, and the potential consequences for bacterial immunity to intracellular pathogens such as Mtb, are critical questions that have not yet been clearly defined. We speculate that deregulation of the cell death pathways could explain, at least in part, the association observed between exposure to CS and pulmonary TB. 

In this study, we tested the hypothesis that macrophage exposure to CS particles triggers intracellular cell death pathways in such a way that those macrophages become more prone to necrosis than apoptosis when infected with Mtb.

## Methods

### Ethics Statement

Blood specimens were acquired from buffy coats by the blood bank personnel at the National Institute of Respiratory DIseases, Mexico City. The study was approved by the Institutional Review Board (IRB# B03-12) of the National Institute of Respiratory Diseases and was conducted following the principles stipulated in the Declaration of Helsinki.

### Preparation of crystalline silica

CS with a median diameter of 5 μm was first heated at 180°C for 2 h to remove all traces of lipopolysaccharides (LPS). 

### Cell culture

#### THP-1 monocytes

The human monocyte cell line THP-1 (American Tissue Type Collection, Rockville, MD, USA) was grown in a humidified atmosphere at 37°C and 5% CO_2_ in RPMI 1640 medium with l-glutamine (2mM, GIBCO, Grand Island, NY, USA), supplemented with 10% heat-inactivated fetal bovine serum (FBS) (GIBCO, Grand Island, NY, USA), 10 mM HEPES, 1mM pyruvic acid (Sigma-Aldrich, Steinheim, Germany), and penicillin/streptomycin (Sigma-Aldrich, Steinheim, Germany). THP-1 human monocytes were differentiated into macrophages by adding phorbol 12-myristate 13-acetate (PMA) (Sigma-Aldrich, Steinheim, Germany) (100 nM/mL) for 3 h at 37°C.

### Enrichment of monocytes

Peripheral blood mononuclear cells (PBMC) were isolated from buffy coats by standard Lymphoprep^TM^ (Accurate Chemical-Scientific, Westbury, NY, USA) gradient centrifugation. Monocytes were enriched using positive selection via magnetic microbeads coated with antibodies to CD14 (Miltenyi Biotech). The CD14+ fraction was analyzed by flow cytometry to determine the purity of the cells. Purified cells were routinely 90-95% of the intended cell type. CD14+ enriched monocytes were plated at 1X10^5^ cells per well in 96-well plates (Costar, Ontario, Canada) with RPMI 1640 medium (GIBCO, Grand Island, NY, USA), supplemented by l-glutamine (2mM; GIBCO, Grand Island, NY, USA), streptomycin, penicillin and 10% heat-inactivated fetal bovine serum (FBS) (GIBCO, Grand Island, NY, USA). After a 7-day incubation period, viable cells were considered to be monocyte-derived macrophages (MDMs) based on their expression profile of CD14, CD68 and the mannose receptor (data not shown). These mature MDMs were then used in the different experiments.

### Multiparametric flow cytometric analysis

To measure the expression of cell surface markers, 106 THP-1 macrophages were stained and analyzed by flow cytometry. Briefly, cells were stained for 20 min at 4°C with fluorochrome-conjugated mAb to CD68, CD80, CD86, TLR2, TLR4, HLA-DR and MMR (CD206) (BioLegend, San Diego, CA). After incubation, cells were washed and re-suspended in staining buffer prior to flow cytometry. Data were collected using a FACS Aria flow cytometer (Becton Dickinson, San Jose, CA) and FACS Diva software (V.6.1), and then analyzed with FlowJo (Tree Star, Inc. Ashland, OR). Typically, 100,000 events were acquired. 

### Bacteria

Mtb strain H37Rv (Mtb-H37Rv) was used in these experiments. Mtb-H37Rv was grown to the log phase in Middlebrook 7H9 broth (Difco, Detroit, MI) and supplemented with 1% glycerol and 10% Middlebrook albumin dextrose catalase enrichment (Difco, Detroit, MI). The bacteria were then harvested and frozen at -80°C in RPMI 1640, 10% fetal bovine serum, and 6% glycerol. Plating serial 10-fold dilutions on 7H10 agar and counting colonies after incubation for 3 weeks determined bacterial CFU. 

### Silica exposure and in vitro infection

MDM and THP-1 macrophages were grown in 24-well plates (5X10^5^ cells/well). The CS stock suspension (1 μg/μl) was made in RPMI-1640 and vortexed prior to dilution with the respective medium to reach the final concentrations. Cell cultures were exposed to CS for 24 h at the following concentrations: 1, 5 and 10 μg/ml. After 24 h, cells were washed twice with RPMI medium and then infected with Mtb-H37Rv at a multiplicity of infection of (MOI) = 1, as described previously [25]. Briefly, Mtb-H37Rv were opsonized for 5 min using RPMI 1640 medium supplemented with 2% human serum, 10% FBS, 0.05% Tween 80, and washed twice in the complete medium without antibiotics. The bacteria were then passed through a 5 μm syringe filter (Millipore, Carrigtwohill, Ireland), counted in a Petroff-Hausser chamber, and added to MDM at the indicated MOI. The duration of infection was 2 h for all experiments. At day 4 post-infection, cells were lysed with a 0.1% solution of saponin for 5 min and the bacteria were enumerated by plating serial dilutions of cell lysates on Middlebrook 7H10 agar plates. Colonies were counted after 21 days. 

### Cytokine analysis

Culture supernatants from the Mtb-infected THP-1 macrophages or MDMs were passed through a 0.2 μM filter to remove any bacteria. Supernatants were assayed for cytokines by a sandwich ELISA, which was conducted in accordance with the manufacturer’s instructions with absorbance recorded at 405 nm on SoftMax Pro ELISA analysis software (Molecular Devices). The IL-1β and TNF-α in the culture supernatants were quantified by comparison with the appropriate recombinant standard (purchased from R&D Systems).

### Detection of apoptosis: TUNEL assay

THP-1 (10^6^ cells) macrophages were grown on 4-well slides (Millicell EZ slide, Millipore) and exposed for 24 h at the following CS concentrations: 1, 5 and 10 μg/ml. After incubation, cells were washed twice with RPMI medium and infected with Mtb-H37Rv, as described previously. The THP-1 macrophages were washed once in PBS before being fixed in 4% paraformaldehyde for 1 h at RT. After an additional wash, cells were permeabilized by adding a permeabilization solution (0.1% Triton X-100, 0.1% sodium citrate in H20) for 5 min on ice, and washed with PBS. DNA strand breaks were labeled by adding the terminal deoxynucleotidyl transferase-mediated dUTP nick end labeling (TUNEL) reaction mixture, according to the manufacturer’s instructions (In Situ Cell Death Detection Kit, Roche Applied Science). The reaction mixture contained terminal deoxynucleotidyl transferase, which adds fluorescein-labeled nucleotide polymers to the free 3’-OH DNA ends of the fragmented DNA. Samples were incubated for 1 h at 37°C. Nuclei were stained with 4',6-diamidino-2-phenylindole (DAPI) (Santa Cruz Biotechnology, Inc., Santa Cruz, CA, USA), and fluorescence intensities were measured under an OLYMPUS IX81 Evolution™ microscope (Bethesda, MD, USA) at excitation and emission wavelengths of 500 and 535 nm for red fluorescence, and 550 and 700 nm for green fluorescence, respectively.

### Western Blot analysis

The THP-1 macrophages (1X10^6^) were exposed to CS for 24 h (1, 5 and 10 μg/ml). After incubation, cells were washed twice with RPMI medium and infected with Mtb-H37Rv (MOI = 1) for 24 hours at 37°C in a humidified atmosphere containing 5% CO_2_. After infection, cells were washed with PBS and lysed in Laemmli buffer. Equal amounts of protein were subjected to Mini-Protean TGK 4-15% gels (Bio-Rad Laboratories, Hercules, CA) and transferred to a 0.2 μm-pore-size Trans-Blot TurboTM PVDF membrane (Bio-Rad Laboratories, Hercules, CA). Western Blot was performed using the following antibodies: caspase 9, cytochrome c, ASK1, and p-38 (dilution: 1:1000), JKN1/2 (dilution 1:2000) (R&D Systems, Minneapolis, MN, USA), Mcl-1 and Bcl-2 (dilution 1:1000) (Cell Signaling Technology, Inc., Danvers, MA, USA). Protein bands were detected by incubating with horseradish peroxidase-labeled antibodies, and visualized with enhanced chemiluminescence reagent (Thermo Scientific, Pierce Biotech., Rockford, IL) using an Imaging System from Bio-Rad (ChemiDoc^TM^ XRS+ System). Band densities were analyzed by densitometry using online IMAGEJ 1.39c software provided by the NIH (http://rsb.info.nih.gov/ij/index.html), as described by Luke Miller (http://www.lukemiller.or/journal/2007/08/quantifying-westernblots-without.html). Each sample was normalized using Glyceraldehyde 3-phosphate dehydrogenase (GAPDH) as a loading control. 

### In vitro assays of necrosis and apoptosis

The THP-1 macrophages (5X10^5^) were exposed to CS for 24 h. After 24 h, some of the cells were infected with Mtb-H37Rv (MOI = 1) for 24 hours at 37°C in a humidified atmosphere containing 5% CO_2_. After infection, cell death was measured using the enzyme-linked immunosorbent assay cell (Cell Death Detection ELISA PLUS; Roche) to quantify cytoplasmic (apoptosis) and extracellular (necrosis) histone-associated DNA fragments, according to the manufacturer’s protocol. At the times indicated after infection, the relative amounts of necrosis or apoptosis were calculated as a ratio of the absorbance of infected macrophages to that of uninfected control macrophages.

### Statistical analysis

Results are expressed as means ± SEM. Data comparisons were performed using one-way ANOVA followed by Dunnett’s post-hoc test for multiple comparisons. Two-tailed unpaired Student’s *t* tests were used to evaluate differences between control and treated groups. (GraphPad Software, Inc., San Diego, CA).

## Results

### Pre-exposure to CS limits control of intracellular bacterial growth of Mtb in MDM and THP-1 macrophages

**Figure 1:**
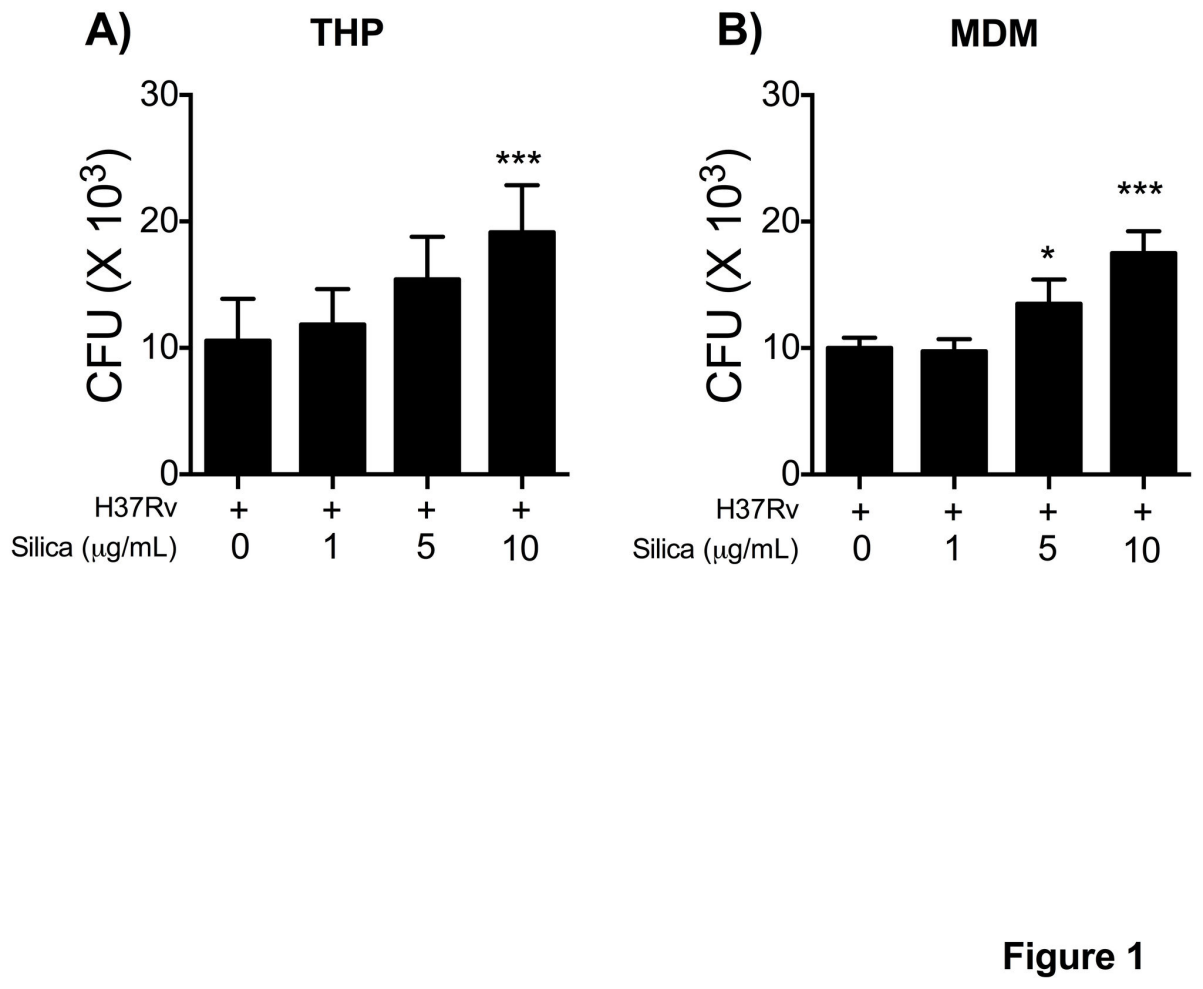
CS limits macrophage ability to control intracellular bacterial growth. THP-1 (A) and MDM (B) macrophages were exposed to CS at concentrations of 1, 5 and 10 μg/ml for 24 h and then infected with Mtb-H37Rv (MOI 1). On day 4 post-infection, CFU were enumerated. Bars indicate mean ± SD from five independent experiments. *P<0.05, **P<0.01. ANOVA and Dunnett’s post-hoc test compared to unexposed macrophages.

Chronic exposure to CS has been associated with pulmonary and extra-pulmonary tuberculosis infection [3,26-28]. The effect of exposing macrophages to CS at concentrations of 1, 5 and 10 μg/ml for 24 h on the bacterial growth of live Mtb is shown in Figure 1A and B. Compared to unexposed macrophages, *in vitro* treatment with CS decreased in a dose-dependent manner the capacity of the macrophages to control the intracellular bacterial replication of Mtb in both THP-1 and MDM macrophages (Figures 1A and B). 

#### CS downregulated TLR2 expression on THP-1 macrophages

THP-1 macrophages have been shown to express a variety of receptors involved in cell activation [29]. Here, the expression of CD68, CD80, CD86, HLA-DR, MMR, TLR2 and TLR4 was analyzed under different experimental conditions (plus or minus exposure to CS, and with or without Mtb-infection). Exposure to CS for 24 h did not modify the expression of CD68, CD80, CD86, HLA-DR or MMR (data not shown); however, when unexposed THP-1 macrophages were infected with Mtb, the frequency and mean fluorescence intensity (MFI) changed, suggesting that under these experimental conditions CS was not responsible for the observed phenotypic changes. 

Activation of the macrophage Toll-like receptors (TLR) 2 and 4-dependent pathway has been described previously after stimulation with coarse particles, and after exposure to TLR ligands such as lipoarabinomannan, phosphatidylinositol mannosides and lipoprotein 19-kDa [30-34]. We ascertained that macrophage exposure to CS downregulated the MFI of TLR2 in a dose-dependent manner (Figure S1-A and -B, supporting information); however, TLR4 expression did not change in response to CS exposure (data not shown). These results suggest that the TLR2 signaling pathway might be altered after macrophage exposure to CS.

### Pro-inflammatory cytokine secretion after macrophage exposure to CS

**Figure 2:**
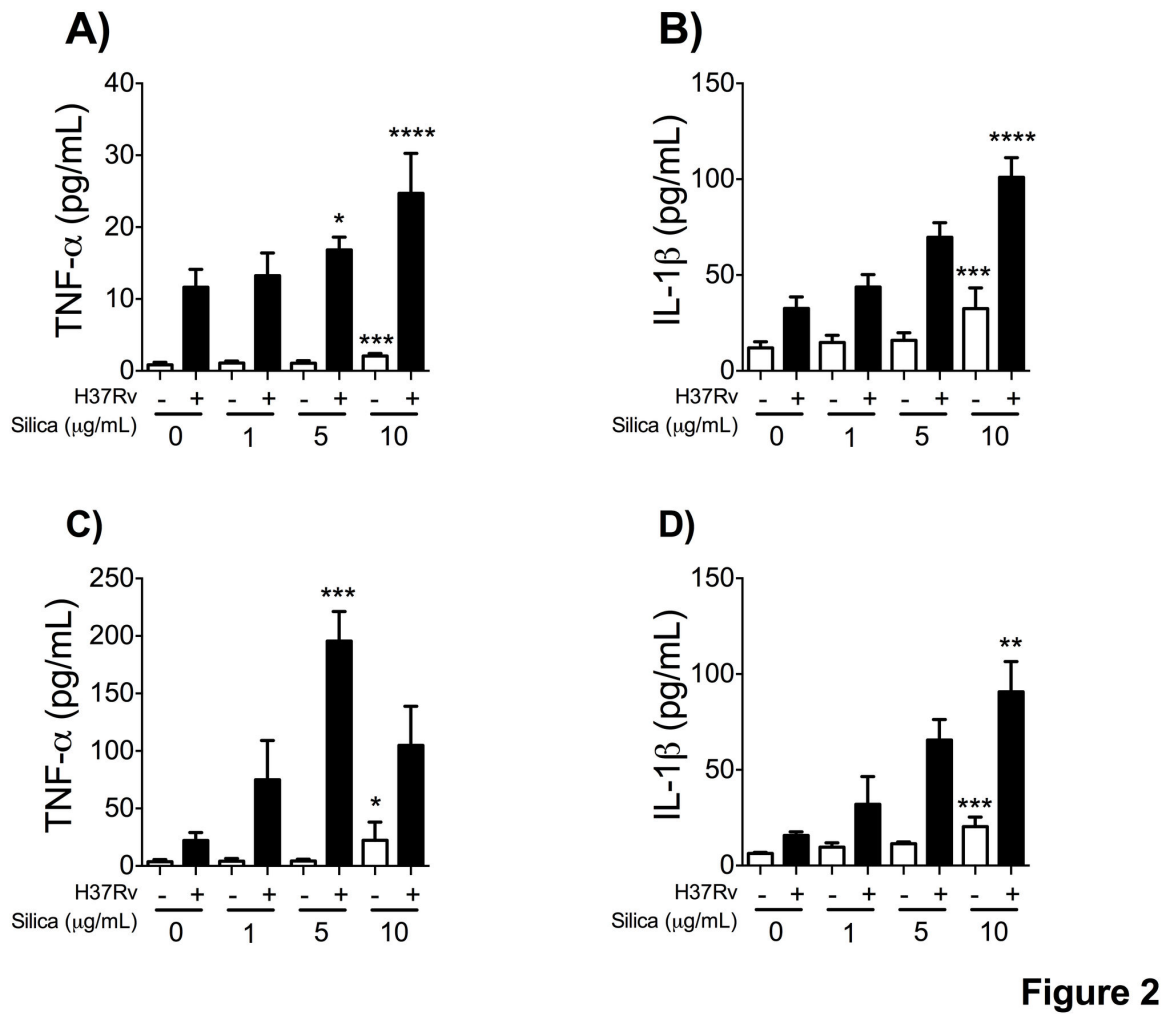
THP-1 and MDM macrophages release pro-inflammatory cytokines after exposure to CS. Concentrations of TNF-α and IL-1β were measured by ELISA in culture supernatant from CS-exposed and unexposed THP-1 (A and B) and MDM (C and D) macrophages infected with Mtb. Bars indicate mean ± SD from eight independent experiments. ****P≤0.0001, **P<0.01, *P<0.05 ANOVA and Dunnett’s post-hoc test compared to unexposed macrophages.

TNF-α and IL-1β are two pro-inflammatory cytokines that participate in macrophage activation and the regulation of cell death pathways during tuberculosis infection [35,36]. To analyze the specificity of CS-induced secretion of pro-inflammatory cytokines, we measured the production of TNF-α and IL-1β in the THP-1 and MDM macrophage culture supernatant. Our results show that macrophages exposed to CS had a minor increased in cytokine production; however, Mtb-H37Rv-infected macrophages had a prominent increase in the secretion of both cytokines, and did so in a dose-dependent manner (Figures 2A-D). 

### Exposure to CS induced cell death of uninfected THP-1 macrophages

The engagement of TLR receptors with mycobacterial ligands on the macrophage surface often leads to not only cytokine secretion but also cell death [37]. Since TLR2 expression was reduced after macrophage exposure to CS, we decided to assess cell death on the macrophages exposed to CS and infected with Mtb. We determined that macrophage exposure to increasing concentrations of CS induced accumulation of TUNEL positive-macrophages (Figure S2, supporting information). TUNEL staining of Mtb-infected THP-1 macrophages demonstrated that the infection alone increased DNA fragmentation when compared with uninfected macrophages; however, we found that CS priming of Mtb-infected macrophages did not directly increase cell death. 

### CS-exposed macrophages increased the expression of ASK1 and JNK1

**Figure 3:**
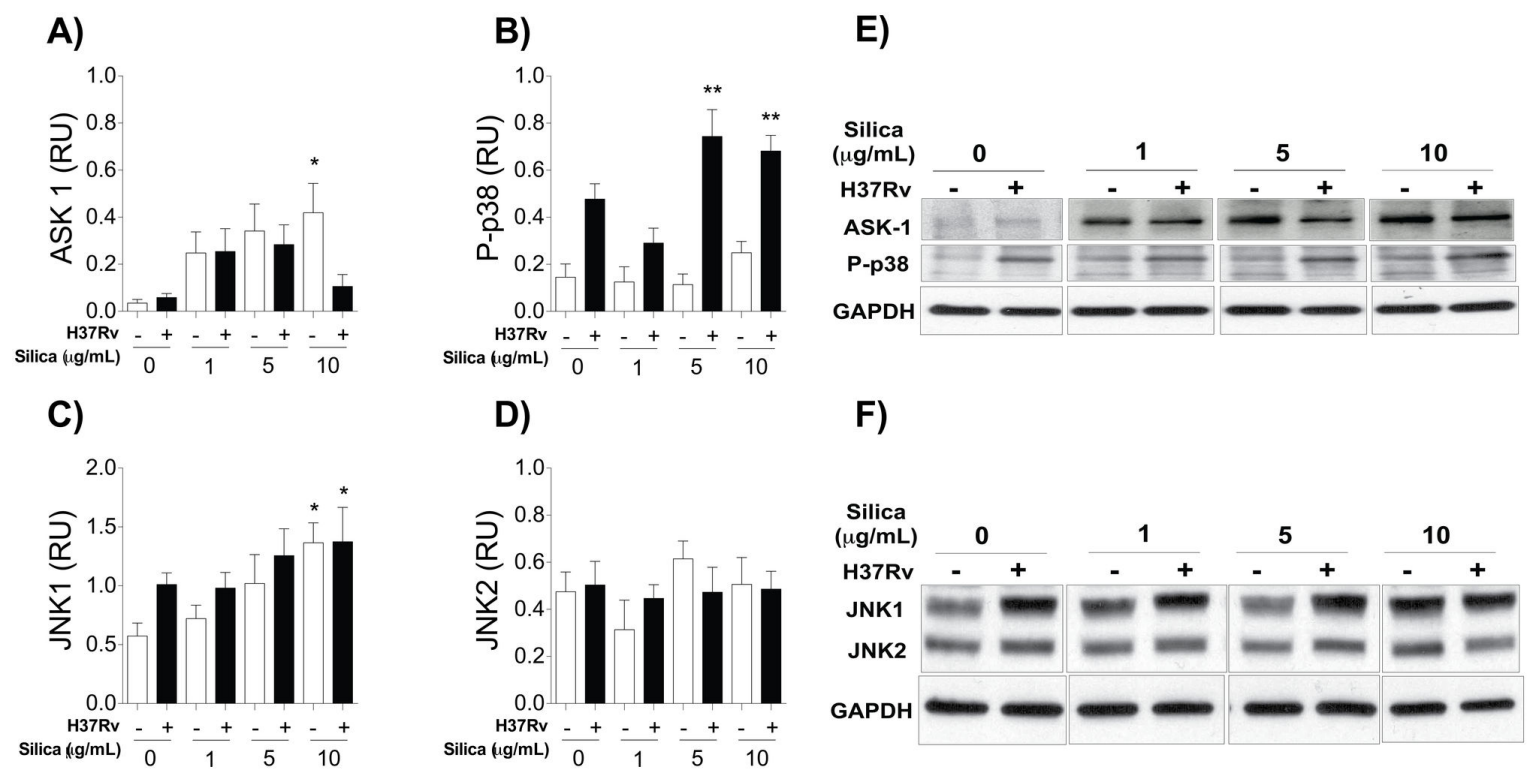
CS increased ASK1 and JNK1 expression in exposed macrophages. THP-1 macrophages were exposed to CS at concentrations of 1, 5 and 10 μg/ml for 24 h and infected with Mtb. Relative units of ASK-1 (A), P-p38 (B), JNK1 (D) and JNK2 (D) are shown; band densities in the Western Blots were normalized against GAPDH. One of the five independent Western Blots for ASK1, P-p38, JNK1 and JNK2 (C, F) is shown. Total protein was determined using GAPDH as a loading control. Bars indicate mean ± SD. *P<0.05, **P<0.01. ANOVA and Dunnett’s post-hoc test compared to unexposed macrophages.

Mitogen-activated protein kinase (MAPK) activity is central to the activation of diverse immunological mechanisms in macrophages. Apoptosis signal-regulating kinase1 (ASK1) is a member of this MAPK kinase family that is activated in response to danger signals [38,39] and, in turn, activates two different subgroups of MAP kinase kinases (MAPKK): JNK (c-Jun amino-terminal kinase) and p38 [40]. Activation of p38 leads to the production and secretion of TNF-α [41], while JNK has been related to numerous cellular mechanisms including development of apoptotic cell death [42,43]. Considering that both cytokine secretion and cell death increased in CS-exposed macrophages, we hypothesized that the MAPK pathway might be involved. To address this possibility, the expressions of ASK1, P-p38, JNK1 and JNK2 were examined by Western Blot. CS induced the expression of ASK1 and JNK1, but not P-p38 (Figure 3A-C). This phenotype was not modified after infection with Mtb. JNK2 showed no significant changes after CS exposure or Mtb-infection (Figures 3D-F). These results agree with our previous findings (Figures 2 and S2), because P-p38 increased in response to both exposure to CS and Mtb-infection, similar to cytokine secretion; while JNK1 increased in response to exposure to CS, similar to cell death. These data suggest that activation of the MAPK pathway and JNK1 occurred in response to CS and might be responsible as one of the potential mechanisms mediating cell death. 

### Exposure to CS decreased levels of anti-apoptotic molecules

**Figure 4:**
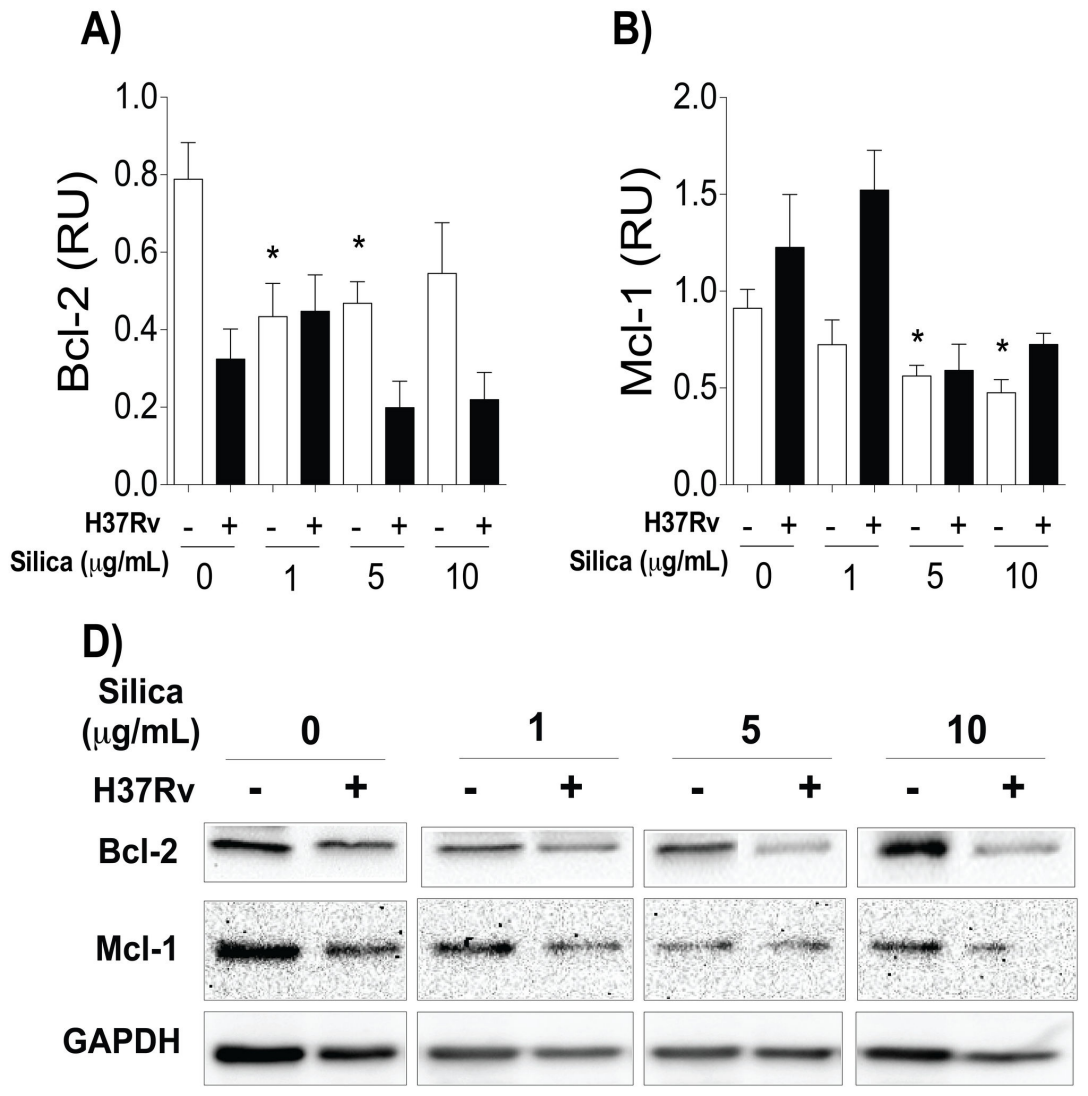
Exposure to CS decreased levels of the anti-apoptotic Bcl-2 and Mcl-1 molecules in macrophages. THP-1 macrophages were exposed to CS at concentrations of 1, 5 and 10 μg/ml for 24 h, then the macrophages were infected with Mtb. Relative units of Bcl-2 (A) and Mcl-1 (B) are shown; band densities in the Western Blots were normalized against GAPDH. One of the four independent Western Blots for Bcl-2 and Mcl-1 (C) is represented. Bars indicate mean ± SD. *P<0.05. ANOVA and Dunnett’s post-hoc test compared to unexposed macrophages.

It is well known that JNK activation may lead to cell death through two different mechanisms: mitochondrial damage or degradation/inactivation of Bcl-2 anti-apoptotic molecules [44-46]. To evaluate whether macrophages exposed to CS downregulate the expression of anti-apoptotic molecules, myeloid cell leukemia-1 (Mcl-1) and B-cell lymphoma-2 (Bcl-2) were measured by Western Blot. We observed that at concentrations of 1 and 5 μg/mL, CS reduced the expression of Bcl-2, while at 10 μg/mL, Mcl-1 expression was significantly reduced (Figures 4A and B). This leads us to think that the reduction of anti-apoptotic molecules is a consequence of JNK activation, and that this particular microenvironment brings about a relative increase in pro-apoptotic molecules and cell death.

### Silica exposure increases expression of caspase 9

**Figure 5:**
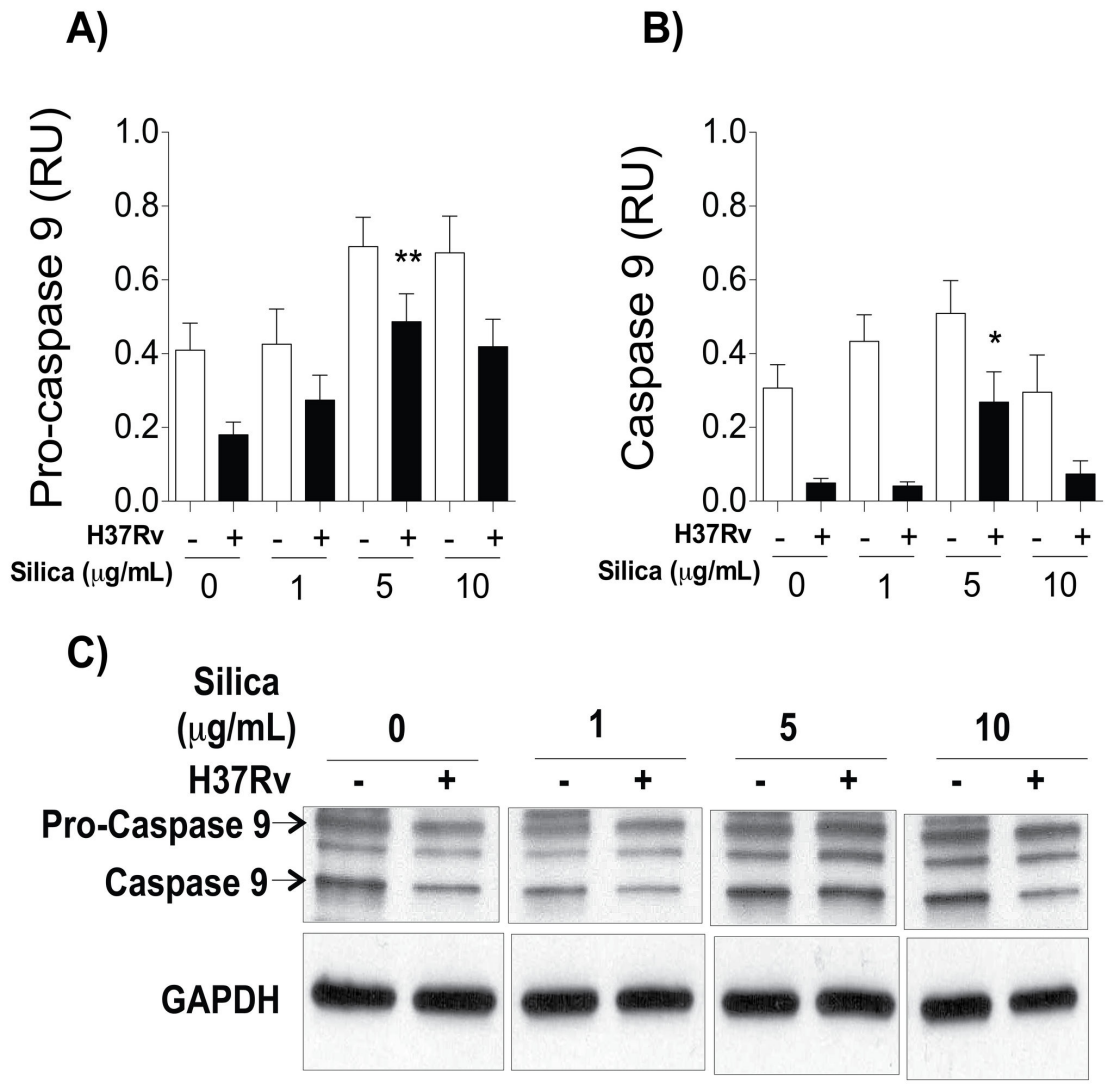
CS increased pro-caspase and caspase 9 levels in macrophages. THP-1 macrophages were exposed to CS at concentrations of 1, 5 and 10 μg/ml for 24 h, then the macrophages were infected with Mtb. Relative units of pro-caspase 9 (A) and caspase 9 (B) are shown; band densities in the Western Blots were normalized against GAPDH. One of the five independent Western Blots for pro-caspase 9 and caspase 9 (C) is represented. Bars indicate mean ± SD. *P<0.05. ANOVA and Dunnett’s post-hoc test compared to unexposed macrophages.

Based on our previous results showing that CS exposure increases ASK1 and JNK expression and reduces Bcl-2 and Mcl-1 molecules, we hypothesized that this phenotype of the macrophage is secondary to mitochondrial damage. In order to test this hypothesis, caspase 9 was measured in uninfected and Mtb-infected macrophages by Western Blot after CS exposure. We observed that macrophages exposed to 5 ug/mL of CS increased the expression of pro-caspase and caspase 9 (Figures 5A and B), suggesting that the signaling pathway leading to cell death might be a consequence of caspase 9 activation and mitochondrial damage. 

### Mtb-infected macrophages are more prone to present necrosis after CS exposure

**Figure 6:**
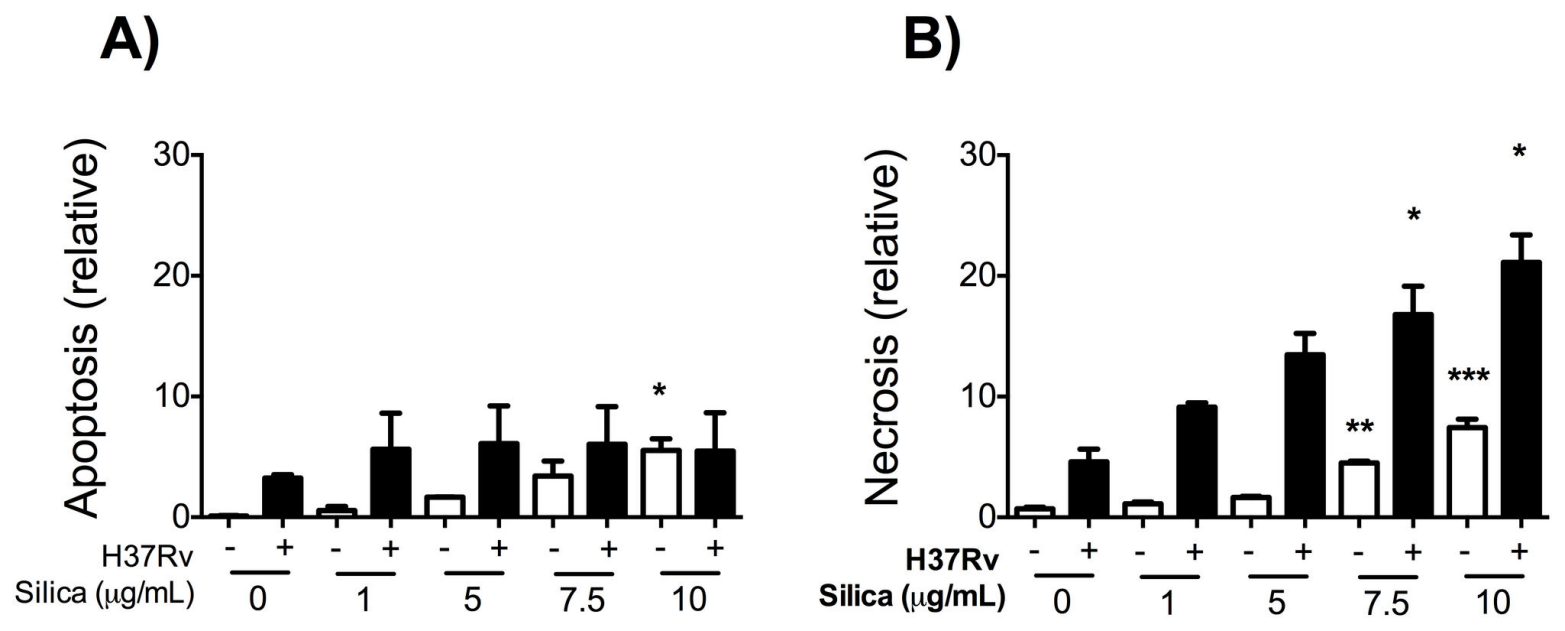
Pre-exposure to CS increased necrosis of Mtb-infected macrophages. THP-1 macrophages were exposed to CS at concentrations of 1, 5 and 10 μg/ml for 24 h, then the macrophages were infected with Mtb. Apoptosis (A) or necrosis (B) was evaluated 24 h after Mtb-infection. Staurosporine (3 μM) was used as a positive control. Data is re_’¡. Bars indicate mean ± SD. *P<0.05. ANOVA and Dunnett’s post-hoc test compared to unexposed macrophages.

We demonstrated previously that CS exposure sensitized macrophages to present cell death as a consequence of the increased expression of ASK1, JNK1 and caspase 9, as well as the degradation of anti-apoptotic molecules. To analyze the possibility that macrophage exposure to CS changes the balance between apoptosis and necrosis, we measured intracellular and extracellular DNA-histone complexes that are indicative of apoptosis and necrosis, respectively [47]. We determined that CS alone increased the frequency of both apoptosis and necrosis in the THP-1 macrophages (Figures 6A and B). When THP-1 macrophages were pre-exposed to CS and later infected with Mtb-H37Rv, the frequency of necrotic cells increased in a dose-dependent manner compared to unexposed infected-THP-1 macrophages. These results demonstrated that when THP-1 macrophages are pre-exposed to CS their steady state profile is changed to a pro-death profile, and that under these modified circumstances infection with Mtb increases the rate of necrosis. 

## Discussion

The most important findings of our study are as follows: 1) CS decreases the capacity of the macrophages to control the intracellular bacterial growth of Mtb; 2) CS induces the secretion of TNF-α and IL-1β; 3) CS increased the intracellular expression of ASK1, JNK1 and caspase 9, but decreased the anti-apoptotic molecules Mcl-1 and Bcl-2; and, 4) pre-exposure to CS increased necrosis of Mtb-infected macrophages. 

*In vitro* exposure of MDM and THP-1 macrophages to CS increased intracellular bacterial growth in a dose-dependent manner (Figures 1A and 1B), suggesting that some mycobactericidal mechanisms of the macrophage were impaired. These results concord with those from Yin, XJ et al., who showed that exposure of AM to diesel exhaust particles (DEP) suppressed the killing of *Listeria monocytogenes* [48]. The reported cellular response of the macrophages exposed to DEP and CS in our study is similar, despite the difference in chemical composition, including the absence of several organic compounds in CS dust. 

Although it has been demonstrated previously that CS downregulates the expression of accessory molecules on dendritic cells [49], we explored whether CS exposure could mediate a similar effect on uninfected and Mtb-infected THP-1 macrophages. We observed no changes in the cell surface expression of the co-stimulatory molecules after macrophage exposure to CS. A discrete change in the expression of CD86, HLA-DR and MMR was observed once cells were infected with Mtb (data not shown); suggesting that 24 h exposure to CS did not lead to macrophage activation.

TLRs are pathogen recognition receptors expressed by several host cells, including monocytes and macrophages. Of these, TLR 2 and 4 have been studied in murine and human in vitro experimental models showing their participation in the innate immune response against Mtb by recognizing pathogen associated molecular patterns to induce cell activation and restriction of intracellular bacterial growth [31,33,34]. Cytometric analysis of THP-1-exposed macrophages revealed that the TLR4 expression level did not change after CS (data not shown); however, TLR2 MFI diminished in a dose-dependent manner (Figure S1-A and -B). These results lead us to suggest that the TLR2 signaling pathway that leads to pro-inflammatory cytokine secretion and cell death might be affected after macrophage exposure to CS.

Nalp3-dependent IL-1β secretion has been described in murine macrophages treated with increasing concentrations of CS [12]. Under our experimental conditions, a small concentration of IL-1β at the different concentrations of CS was observed, but when exposed macrophages (MDM or THP-1) were infected with Mtb we did identify a dose-dependent increase in IL-1β secretion (Figures 2B and 3D). A similar result was noted when TNF-α was analyzed (Figures 2A and 3C). Secretion of these two pro-inflammatory cytokines may participate in the activation of diverse intracellular cell death pathways that are necessary for the immune response against Mtb [1,50,51]. Due to the related concepts that the TLR2-dependent pathway leads to cell death, and that necrosis is considered an evasion mechanism induced by Mtb [52,53], we ascertained that exposure of THP-1 macrophages to 5 μg/ml of CS increased the percentage of TUNEL+ cell (Figure S2). Our data demonstrated that exposure to CS, even at low concentrations, affects the TLR2 activation pathway as it increased both cytokine secretion and cell death (Figures 2 and S2). We selected a low concentration of CS due to previous observations in our laboratory showing that more than 50% of the macrophages exposed to 20 μg/ml or more of CS presented cell death (data not shown). This high rate of cell death could bias our results and the interpretation we were addressing.

MAPK kinase activity is induced after Mtb interaction with macrophages or by the expression of danger signals [40,54]. In our experimental model we identified that CS exposure increased the expression of ASK1 and JNK1 levels in a dose-dependent manner (Figures 3A and 5D). Expression of p38 increased only after Mtb infection (Figure 5B): a result that concords with the cytokine expression profile we found previously (Figure 2). Similar to previous findings, we identified that macrophage exposure to CS leads to the intracellular activation of at least two molecules of the MAPK pathway, ASK1 and JNK1 [55]. We observed that CS exposure increased JNK1 expression and diminished Bcl-2 and Mcl-1 molecules (Figures 4A and 6B). JNK1 signaling pathway is responsible for Bcl-2 multi-site phosphorylation/inactivation and Bcl-2 has been studied most extensively as an anti-apoptotic molecule [56]. Early, transient JNK1 activation promotes cell survival whereas prolonged JNK1 activation can mediate apoptosis [57].

**Figure 7:**
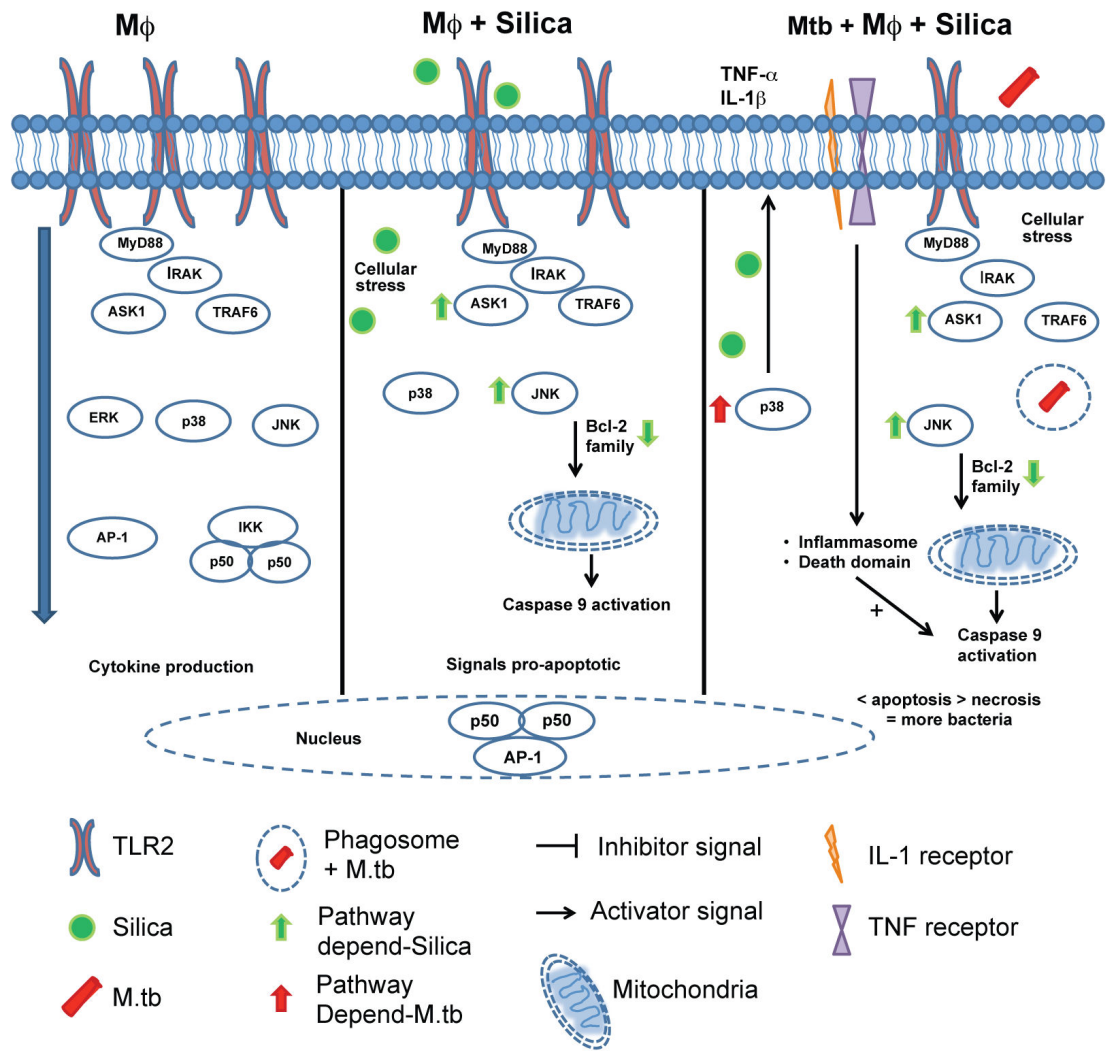
Proposed model for immunological damage in Mtb-H37Rv-infected macrophages pre-exposed to CS. In normal conditions, TLR2 activation in a dose-dependent manner leads to cytokine production in the macrophages. 2) Macrophages exposed to CS have increased ASK1 and JNK1 levels (perhaps as signals of cellular stress), but decreased expression of TLR2 and levels of the anti-apoptotic molecules Bcl-2 and Mcl-1; under these conditions, the macrophage is more susceptible to cell death. 3) Where the macrophages pre-exposed to CS are infected with Mtb-H37Rv, there is an increased secretion of pro-apoptotic cytokines (TNF-α and IL-1β). When macrophages are exposed to CS, they become more susceptible to cell death, if they are also infected with M.tb, pro-inflammatory cytokines are secreted and necrosis is established, favoring the release of viable intracellular bacilli.

We thus speculated that macrophages exposed to CS are more prone to cell death than unexposed macrophages. To elucidate whether this pro-apoptotic profile leads to mitochondrial damage and activates signaling pathways associated with cell death, we measured pro-caspase and caspase 9 (Figures 5A and 7B). Our data confirmed that macrophages exposed to CS were more prone to cell death than controls because they had increased caspase 9, possibly due to mitochondrial damage. 

Finally, to demonstrate that CS deregulates the critical balance of apoptosis *vs.* necrosis in the cellular pathways that participate in the immune response against Mtb, we measured intracellular and extracellular DNA-histone complexes in exposed macrophages, finding that CS increased apoptosis in uninfected macrophages but increased necrosis in a dose-dependent manner in those infected with Mtb (Figure 6B). This constitutes a detrimental effect on host defenses as it favors the release of viable intracellular bacilli and, therefore, the progression of tuberculosis. This change is produced by an imbalance in the MAPK activation pathway that culminates in cytokine production and macrophage apoptosis.

## Conclusion

We propose, for the first time, a mechanism (Figure 7) through which exposure to CS affects the capacity of the macrophages to control the intracellular bacterial replication of Mtb. TLR2-dependent signaling leads to activation of the MAPK kinase pathway, thus inducing mainly cytokine secretion or cell death. When macrophages are exposed to CS, decreased TLR2 expression and increased ASK1 levels result. ASK1 activation induces increasing levels of JNK1 and the anti-apoptotic Caspase 9 molecules are activated. This imbalance in the MAPK kinase pathway makes the macrophages more susceptible to cell death. When pre-exposed macrophages are infected with Mtb, secretion of pro-apoptotic cytokines (TNF-α and IL-1β) increases. Together, the imbalance of anti-apoptotic molecules plus mitochondrial damage leads to a high rate of macrophage necrosis.

## Supporting Information

Figure S1 **Macrophages exposed to CS decreased TLR2 expression.** THP-1 macrophages were exposed to CS at concentrations of 1, 5 and 10 μg/ml for 24 h and then infected with Mtb-H37Rv. The macrophages were harvested and stained with mAb against TLR2. The frequency and MFI of TLR2 expression (A and B) between the uninfected and infected macrophages were analyzed. Bars indicate mean ± SD from five independent experiments. *P<0.05. ANOVA and Dunnett’s post-hoc test compared to unexposed macrophages.
(TIFF)

Figure S2 **CS induces cell death of uninfected macrophages.** THP-1 macrophages were exposed to CS at concentrations of 1, 5 and 10 μg/ml for 24 h and then infected with Mtb. The graph shows the percentage of THP-1 macrophages that were positive for TUNEL staining. Bars indicate mean ± SD from five independent experiments. *P<0.05. ANOVA and Dunnett’s post-hoc test compared to unexposed macrophages.
(TIFF)
