# Supplementary figures and images for: Pre-Exposure of Mycobacterium tuberculosis-Infected Macrophages to Crystalline Silica Impairs Control of Bacterial Growth by Deregulating the Balance between Apoptosis and Necrosis
Source: PLoS One. 2013 Nov 22;8(11):e80971. doi: 10.1371/journal.pone.0080971 (PMC3838437; doi:10.1371/journal.pone.0080971)

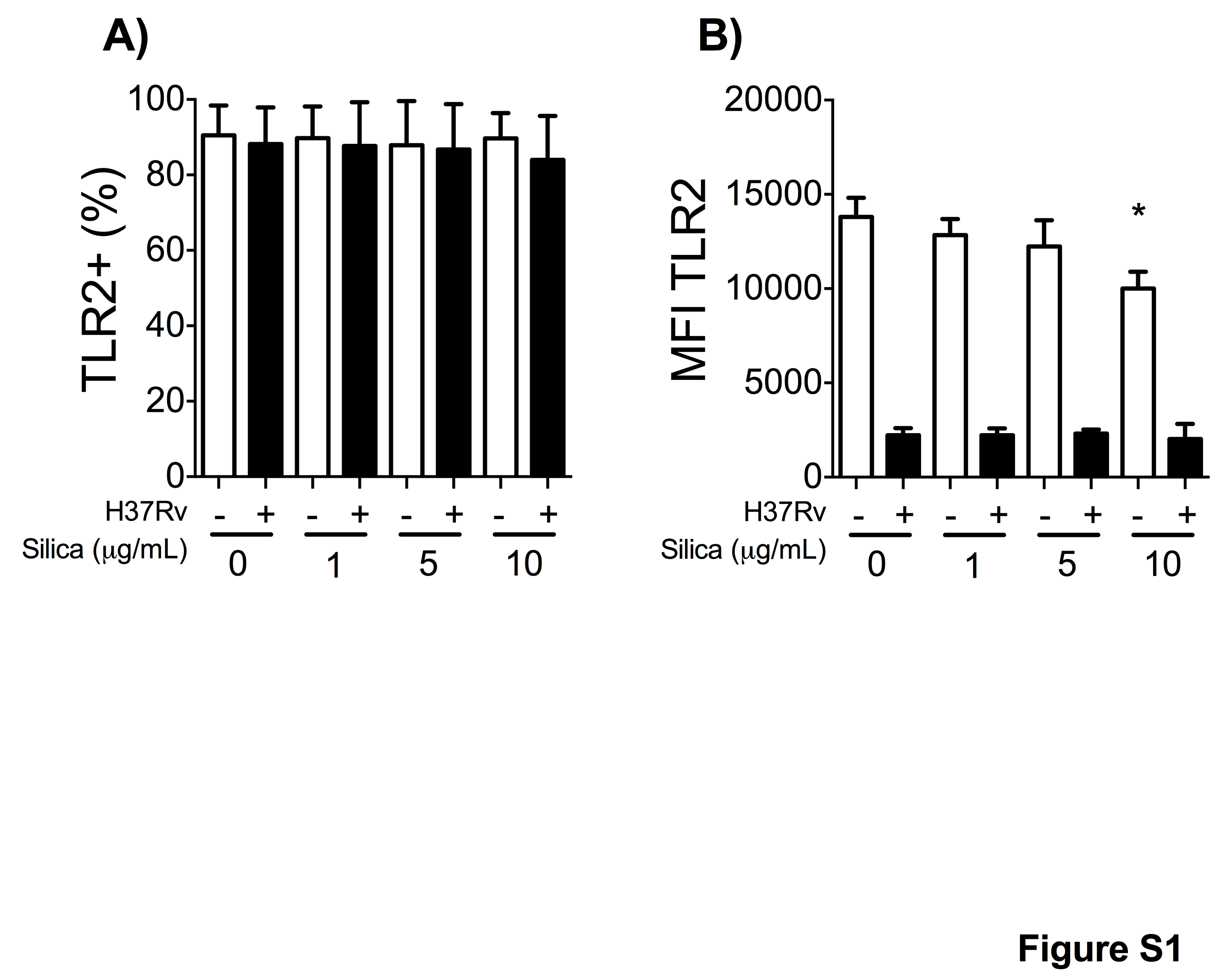

Supplement: Figure S1 — Macrophages exposed to CS decreased TLR2 expression. THP-1 macrophages were exposed to CS at concentrations of 1, 5 and 10 μg/ml for 24 h and then infected with Mtb-H37Rv. The macrophages were harvested and stained with mAb against TLR2. The frequency and MFI of TLR2 expression (A and B) between the uninfected and infected macrophages were analyzed. Bars indicate mean ± SD from five independent experiments. *P<0.05. ANOVA and Dunnett’s post-hoc test compared to unexposed macrophages. (TIFF) [file pone.0080971.s001.tiff]

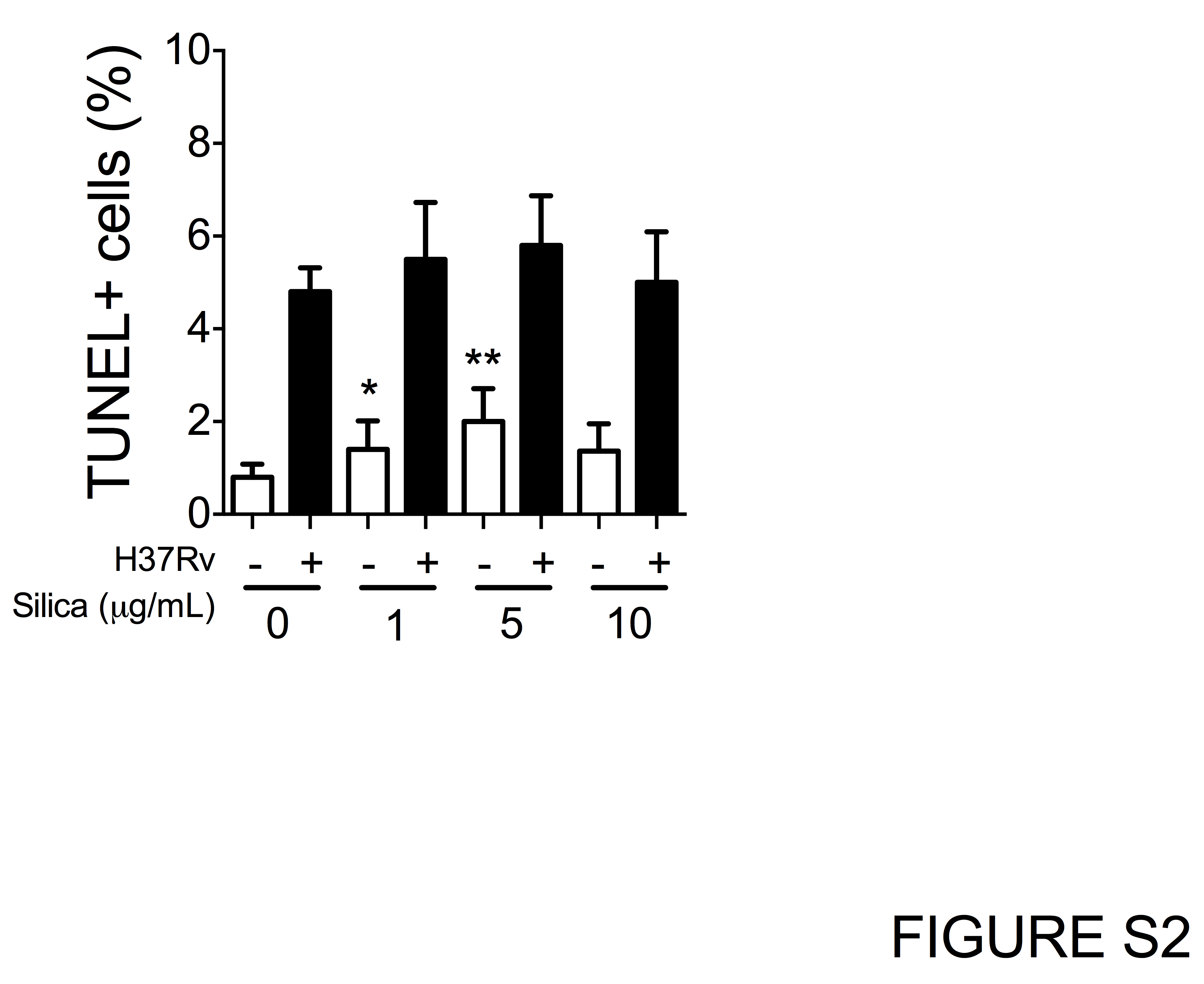

Supplement: Figure S2 — CS induces cell death of uninfected macrophages. THP-1 macrophages were exposed to CS at concentrations of 1, 5 and 10 μg/ml for 24 h and then infected with Mtb. The graph shows the percentage of THP-1 macrophages that were positive for TUNEL staining. Bars indicate mean ± SD from five independent experiments. *P<0.05. ANOVA and Dunnett’s post-hoc test compared to unexposed macrophages. (TIFF) [file pone.0080971.s002.tiff]
